# Investigation of 23 Bile Acids in Liver Bile in Benign and Malignant Biliary Stenosis: A Pilot Study

**DOI:** 10.1155/2019/5371381

**Published:** 2019-12-18

**Authors:** Stanislav Rejchrt, Milos Hroch, Rudolf Repak, Tomas Fejfar, Tomas Douda, Darina Kohoutova, Eva Peterova, Jan Bures

**Affiliations:** ^1^2nd Department of Internal Medicine-Gastroenterology, Charles University, Faculty of Medicine in Hradec Kralove and University Hospital, Sokolska 581, 500 05 Hradec Kralove, Czech Republic; ^2^Department of Medical Biochemistry, Charles University, Faculty of Medicine in Hradec Kralove, Zborovska 2089, 500 03 Hradec Kralove, Czech Republic; ^3^The Royal Marsden Hospital NHS Foundation Trust, Fulham Road, Chelsea, SW3 6JJ London, UK

## Abstract

Differential diagnosis between benign and malignant biliary stenosis can be difficult in clinical practice. Histology of biopsy specimens is often indeterminate. Laboratory markers (serum bilirubin > 75 *μ*mol/L, carbohydrate antigen 19-9 > 400 U/mL) and the length of stenosis (>15 mm) can be helpful but are not specific enough. The aim of this study was to investigate bile acids in liver bile of patients with benign and malignant biliary stenosis and controls without stenosis. A total of 73 patients entered the study: 7 subjects with benign biliary stenosis (6 men, 1 woman; 68 ± 13 years old), 21 with malignant biliary stenosis (15 men, 6 women; 72 ± 14 years old), and 45 patients without biliary stenosis (22 men, 23 women; 70 ± 13 years old); out of those, 25 subjects have and 20 do not have choledocholithiasis. Twenty-three different bile acids were investigated by high-performance liquid chromatography/mass spectrometry. Serum total bilirubin was significantly higher in patients with malignant biliary stenosis compared with nonstenotic controls (*p* = 0.005). Significant relationship (*r* > 0.7) was found between several pairs of bile acids. Significantly lower bile acid concentrations in malignant biliary stenosis compared to controls without stenosis were found for GLCA (*p* = 0.032), GUDCA (*p* = 0.032), GCDCA (*p* = 0.006), GDCA (*p* = 0.031), GHCA (*p* = 0.005), TUDCA (*p* = 0.044), and TDCA (*p* = 0.036). Significant difference in cholic acid was found between benign and malignant stenosis (*p* = 0.022). Analysis of bile acids might be helpful in the differential diagnosis of malignant and benign biliary stenosis. More patients need to be enrolled in further studies so that the real diagnostic yield of bile acids can be determined.

## 1. Introduction

Differential diagnosis of benign and malignant biliary stenosis can be difficult in the clinical practice. Histology of biopsy specimens is often indeterminate. Laboratory markers (serum bilirubin > 75 *μ*mol/L, carbohydrate antigen 19-9 > 400 U/mL) and the length of stenosis (>15 mm) can be helpful but are not specific enough [1]. One study of altered bile composition in benign biliary strictures (after liver transplantation) has been published [2]. Few other studies evaluated bile acids in patients with cancer in the biliary tree [3–5]. Other authors looked at the biomarkers which can help in differentiation between benign and malignant biliary stenosis in other body fluids for the sample matrix [6–8]. The biological basis of benign and malignant biliary stenosis differs significantly. One can assume that different compositions and/or concentrations of bile acids can impact on these processes. The aim of our study was to evaluate 23 different bile acids in liver bile of consecutive patients to analyze whether patients with malignant biliary stenosis have a different concentration of bile acids from the concentration of bile acids of patients with benign stenosis or without stenosis. This observation could have relevance in the differential diagnosis.

## 2. Materials and Methods

### 2.1. Patients

A total of 73 patients entered the study: 7 subjects with benign biliary stenosis (6 men, 1 woman; 68 ± 13 years old), 21 with malignant biliary stenosis (15 men, 6 women; 72 ± 14 years old), and 45 patients without biliary stenosis (22 men, 23 women; 70 ± 13 years old); out of those, 25 subjects have and 20 do not have choledocholithiasis. Seven patients received long-term treatment with peroral UDCA (daily dose 500-1500 mg; median 500, IQR 500-875).

### 2.2. Sample Collection and Storage

Serum total bilirubin and C-reactive protein were investigated less than 48 hours before ERCP (endoscopic retrograde cholangiopancreatography). Samples of hepatic bile were collected from the common hepatic duct endoscopically (prior to contrast media use) and frozen at -80°C immediately until the analysis was carried out.

### 2.3. Chromatography and Mass Spectrometry

#### 2.3.1. Chromatography

The separation of 23 endogenous bile acids was done using UPLC system Acquity I-Class. Briefly, the separation was carried out on an Acquity BEH C18 column (50 × 2.1 mm with 1.7 *μ*m particle size) (Waters, Milford, USA), protected with a disposable 0.2 *μ*m in-line filter (Waters, Milford, USA). The separation was performed in a gradient elution mode with a flow rate of 0.35 mL min^−1^ and with the following composition of mobile phases: Solvent A consisted of ammonium acetate (0.5 mM) and acetic acid (0.001% *v*/*v*) in water. Solvent B was composed of methanol : acetonitrile (75 : 25, *v*/*v*) mixture containing 0.5 mM ammonium acetate and 0.001% (*v*/*v*) of acetic acid. The gradient program was as follows: 0-0.2 min, 40% of solvent B; 0.2-7.0 min, 40-70% of solvent B; 7.0-8.0 min, 70-90% of solvent B; 8.0-8.5 min, 90 - 95% of solvent B; and 9.0-11 min, 40% of solvent B.

The column was held at 45°C during the analysis. Samples were kept at 10°C in a light-tight autosampler unit. Two microlitres of sample was injected into the column. Retention times of all 23 bile acids were verified with authentic standards, and quantification was done against the 8-point calibration curve covering range 5-2000 nmol L^−1^.

#### 2.3.2. Mass Spectrometry

A Xevo-TQ/XS mass spectrometer (Waters, Milford, USA) coupled with the Acquity I-Class UPLC was used for detection of compounds. The spectrometer was operated with an ESI interface in a negative ion mode. Optimized settings of an ion source were as follows: capillary voltage, 2.5 kV; cone voltage, 50 V; source temperature, 150°C, desolvation temperature, 600°C; desolvation gas, 1000 L/h; cone gas, 150 L/h; and nebuliser, 6 L/h.

Compounds were monitored using the multiple reaction monitoring (MRM) mode of operation. Since nonconjugated bile acids generally do not offer CID (collision-induced dissociation) fragments of sufficient intensity for quantification, the so-called pseudo-MRM was used for their quantification. The following MRM transitions were used: 375 ⟶ 375 (nonconjugated, monohydroxy BA), 391 ⟶ 391 (nonconjugated, dihydroxy BA), 407 ⟶ 407 (nonconjugated, trihydroxy BA), 432 ⟶ 74 (glycine-conjugated, monohydroxy BA), 448 ⟶ 74 (glycine-conjugated, dihydroxy BA), 464 ⟶ 74 (glycine-conjugated, trihydroxy BA), 482 ⟶ 80 (taurine-conjugated, monohydroxy BA), 498 ⟶ 80 (taurine-conjugated, dihydroxy BA), and 514 ⟶ 80 (taurine-conjugated, trihydroxy BA). Energies involved in CID were as follows: nonconjugated bile acids, CE = 10 eV; glycine-conjugated bile acids, CE = 30 eV; and taurine-conjugated bile acids, CE = 60 eV. MassLynx and TargetLynx software was used for LC/MS data acquisition and evaluation (version 4.2, Waters, Milford, USA).

#### 2.3.3. Sample Preparation for LC/MS Analysis

Due to high concentration of bile acids in bile, the samples were diluted prior to analysis. Two dilution factors, 1000x and 10000x, were used to cover a broad range of concentrations, including major and minor bile acids in the samples.

A sample preparation corresponding to the 1000x dilution factor was as follows: Ten microlitres of bile in the 1.5 mL Eppendorf tubes was diluted with 90 *μ*L of 0.1% HCOOH in 50% (*v*/*v*) acetonitrile. In the next step, 900 *μ*L of acetonitrile solution containing 1% (*v*/*v*) of formic acid and internal standard IS-CAD_5_ (*c* = 5 *μ*M) was added to the same tube.

The samples with the dilution factor 10000x were prepared as follows: Ten microlitres of bile in the 1.5 mL Eppendorf tubes was diluted with 990 *μ*L of 0.1% (*v*/*v*) formic acid in 50% (*v*/*v*) acetonitrile. One hundred microlitres of this solution was taken and pipetted to a clean 1.5 mL Eppendorf, to which 900 *μ*L of cold acetonitrile solution containing 1% (*v*/*v*) of formic acid and internal standard IS-CAD_5_ (*c* = 5 *μ*M) was further added.

The samples, with the dilution factor 1000x or 10000x, prepared in the previous step were vortexed for 1 minute, left for 10 minutes at -20°C to achieve sample deproteination, and spun at 14.000 × *g* (5 min, room temperature). One hundred microlitres of a supernatant was taken and mixed in a clean 1.5 mL Eppendorf tube with 900 *μ*L of 0.1% (*v*/*v*) formic acid in 50% (*v*/*v*) acetonitrile. Samples were vortexed, and 250 *μ*L is transferred to the AcroPrep filter plate (96 wells, wwPTFE membrane, pore size 0.20 *μ*m, Pall Corporation, USA). The filtrate was collected to a 96-well sample collection plate (700 *μ*L, round well, Waters, Milford, USA), sealed with a sealing mat (Cap-mat 96-well 7 mm round plug preslit silicone/PTFE, Waters, Milford, USA), and put into an autosampler for analysis.

### 2.4. Statistical Analysis

The obtained data were tested statistically by means of descriptive statistics. Data with normal distribution were further analyzed by the parametric unpaired *t*-test, and data with nonnormal distribution were tested by the nonparametric Mann-Whitney test. The Pearson test was used for correlation analysis (SigmaStat software, version 3.1, Jandel Corp., Erkrath, Germany).

### 2.5. Ethics

The study was approved by the Joint University Ethics Committee (Protocol No. 201712S06P). All procedures were in accordance with the ethical standards of the institutional research committee and with the 1964 Helsinki declaration and its later amendments.

All patients signed the written consent. For all data obtained, all personal identification information was deleted in compliance with the laws for the protection of confidentiality of the Czech Republic.

## 3. Results

**Figure 1:**
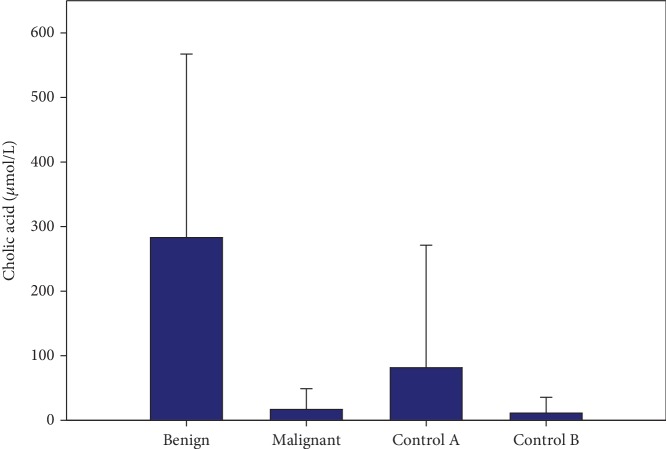
Bile cholic acid in benign (*n* = 7) and malignant (*n* = 21) biliary stenosis and controls without stenosis with (control A; *n* = 25) or without (control B; *n* = 20) choledocholithiasis (mean + Std.Dev.). Significant difference between benign and malignant stenosis was found (*p* = 0.022).

Serum total bilirubin was significantly higher in patients with malignant biliary stenosis compared with nonstenotic controls (*p* = 0.005; see Table 1). Significant relationship (*r* > 0.7) was found between several pairs of bile acids (GLCA-GDCA, GLCA-TLCA, GCDCA-GDCA, GCDCA-GCA, CDCA-CA, GUDCA-TUDCA, GDCA-TDCA, TCDCA-TCA, and TMCA-THCA). Significantly lower bile acid concentrations in malignant biliary stenosis compared to controls without stenosis were found for GLCA (*p* = 0.032), GUDCA (*p* = 0.032), GCDCA (*p* = 0.006), GDCA (*p* = 0.031), GHCA (*p* = 0.005), TUDCA (*p* = 0.044), and TDCA (*p* = 0.036). Significant difference between benign and malignant stenosis was found for cholic acid (CA) (*p* = 0.022, Figure 1). Other results did not reach statistical significance. Concentrations of serum C-reactive protein and 15 bile acids in primary bile in the investigated groups are listed in Table 1. The remaining 8 bile acids were not detectable or their concentrations were <2.5 *μ*mol/L. Total bile acid concentrations are listed in Table 1. They were higher in malignant stenosis compared to benign stenosis (*p* = 0.027) and controls without stenosis (*p* < 0.001).

Bile UDCA was detectable in 14/73 persons (19%). Out of those, 7 patients were not treated and 7 subjects were treated with peroral UDCA (daily dose 500-1500 mg; median 500, IQR 500-875). Bile UDCA was lower in the nontreated group (40.0 ± 44.7 vs. 186.2 ± 182.9 *μ*mol/L), yet this difference did not reach statistical significance (*p* = 0.128, type 2 error beta 0.632). Bile UDCA did not correlate tightly enough either with bile GUDCA (*r* = 0.513, *p* < 0.001) or with bile TUDCA (*r* = 0.388, *p* < 0.001). Correlation between bile GUDCA and bile TUDCA was significant (*r* = 0.835, *p* < 0.001). Seven patients treated with peroral UDCA had significantly higher concentrations of bile GUDCA (*p* < 0.001) and bile TUDCA (*p* < 0.001) compared to those without treatment with peroral UDCA. Treatment with peroral UDCA did not influence significantly the concentration of other bile acids in primary liver bile.

## 4. Discussion

Our current pilot case-control study has shown a new original insight. Bile acid concentrations were significantly lower in malignant biliary stenosis compared to controls without stenosis. There was a significant difference of cholic acid between benign and malignant stenosis. The number of analyzed bile acids is unique. Such a detailed analysis might enable precise mapping of different pathological situations.

One study of altered bile composition in benign biliary strictures (after liver transplantation) was published [2]. Bile salts, phospholipids, and cholesterol were significantly lower in 14/111 patients (13%) who developed nonanastomotic biliary strictures after liver transplantation. Patients who developed biliary stenosis were characterized by a reduced biliary secretion of bile salts and phospholipids and a decreased biliary phospholipid/bile salt ratio. Four bile salts were investigated (cholate, deoxycholate, chenodeoxycholate, and ursodeoxycholate): there were no significant differences in the absolute amounts of the various bile salts between patients who later developed and those who have not developed nonanastomotic biliary strictures [2].

Few other studies analyzed bile acids in patients with cancer of the biliary tree [3–5]. Lankisch et al. [4] published a bile proteomic analysis enabling differentiation of cholangiocarcinoma from primary sclerosing cholangitis and choledocholithiasis. Subsequently, a US patent was submitted [9]. Voigtländer et al. from the same research group [10] published a paper on early diagnosis of cholangiocarcinoma. In this study, combined bile and urine proteome analysis was first established on 87 patients with known diagnosis and then applied to a group of 45 patients without established diagnosis at the time of sample analysis. Thus, validation of the diagnostic method was carried out in a prospective study cohort [10]. Park et al. [3] analyzed five bile acids (cholic, deoxycholic, chenodeoxycholic, lithocholic, and ursodeoxycholic acid) in the biliary tract cancer group, bile duct stone group, and controls. Total bile acid concentration was lower in the cancer group than in the biliary stone and control groups. This result was similar when disease localisation was limited to the bile duct or gallbladder. Although the presence of bile duct obstruction explains some of the differences in total concentration and composition of bile acids, there are other contributing mechanisms. Park et al. stated that alteration of bile acid transport might decrease bile acid excretion and can lead to accumulation of carcinogenic bile acids in the bile duct epithelium [3].

A key question that must be addressed is what is the cause and what is the consequence. Bile salts are potentially cytotoxic [11, 12] and antiapoptotic and might cause cholangiocyte injury and induce periductal fibrosis [13]. Bile acids due to their signalling properties have differential effects on cholangiocyte intracellular regulation, and they can initiate inverse effects on cholangiocyte secretion, proliferation, and survival; e.g., taurocholic and taurolithocholic acids can stimulate, whilst ursodeoxycholic acid may reduce the proliferative effect of other bile acids [2, 14–16]. In an experimental study in rats, intrahepatic accumulation of bile acids did not induce carcinogenesis directly but facilitated a cocarcinogenic effect due to stimulation of bile duct proliferation, enhanced inflammation (increased expression of interleukin-6), and reduction in farnesoid X receptor-dependent chemoprotection (downregulation of FXR) [17].

Although the pharmacokinetics of UDCA have been studied since its approval for its use in primary biliary cholangitis, limited work has been published on modelling the metabolism of UDCA and its two major conjugates, a glycine conjugate, GUDCA, and a taurine conjugate, TUDCA [18]. In our study, seven patients treated with peroral UDCA had significantly higher concentrations of bile GUDCA and bile TUDCA.

Our current project is a pilot study, and our data must be interpreted with caution. We are not able to provide any final explanation for the difference found. Nevertheless, we can hypothesize that long-standing low concentrations of cholic acid can contribute to biliary mucosal instability as one of initial steps in tumorous biology. Unlike us, Jusakul et al. [5] investigated the bile acid profile in gallbladder bile from patients who underwent liver resection for cholangiocarcinoma, hepatocellular carcinoma, and benign biliary diseases. They found markedly elevated cholic and chenodeoxycholic acid in cholangiocarcinoma with high total serum bilirubin. The authors revealed a different pattern of bile acid concentration in cancer patients compared to patients with benign biliary diseases. Accumulation of certain bile acids may be involved in carcinogenesis according to these authors [5].

We are aware of possible limits of this pilot study. The number of patients was limited, especially those with benign stenosis. The control group was rather heterogenic, with or without previous cholecystectomy, some patients underwent repeated ERCPs with or without papillotomy, common bile duct stones were of different aetiology, and not all bile samples were sterile. Patients with malignant stenosis and subjects without stenosis and without choledocholithiasis have both low levels of cholic acid. This observation does not rule out the possible utility of concentration of cholic acid in differential diagnosis between benign and malignant stenosis.

## 5. Conclusions

Analysis of bile acids might be helpful in the differential diagnosis of malignant and benign biliary stenosis. More patients must be enrolled in the future studies so that the real diagnostic yield of bile acids can be determined.

## Figures and Tables

**Table 1:** Serum bilirubin and C-reactive protein and bile acids in liver bile in benign and malignant biliary stenosis and controls without stenosis.

Parameter	Benign stenosis (*n* = 7) Mean ± Std.Dev. C.I. of mean Median IQR	Malignant stenosis (*n* = 21) Mean ± Std.Dev. C.I. of mean Median IQR	Controls without stenosis (*n* = 45) Mean ± Std.Dev. C.I. of mean Median IQR
Serum bilirubin (*μ*mol/L)	39.4 ± 47.9	174,667 ± 178,556	37.4 ± 42.9
	44.3	81.3	12.9
	15.0	138.0	15.0
	11.0–49.0	14.5–310.0	11.0–49.0

C-reactive protein (mg/L)	15.1 ± 27.8	40.7 ± 50.8	31.8 ± 42.6
	25.7	28.1	14.2
	2.5	10.0	11.0
	2.0–13.3	5.5–57.5	2.8–55.0

CDCA (*μ*mol/L)	203.4 ± 305.4	18.3 ± 25.1	68.7 ± 157.9
	282.4	11.4	47.4
	43.8	2.9	16.6
	4.5–269.7	0–27.9	0–29.5

GLCA (*μ*mol/L)	73.1 ± 56.9	38.3 ± 62.8	169.6 ± 272.6
	52.6	28.6	81.9
	73.0	12.4	32.1
	27.2–105.8	0–44.7	4.1–260.1

GUDCA (*μ*mol/L)	3003.2 ± 4860.6	740.5 ± 1847.6	2160.1 ± 5088.9
	4495.4	841.0	1528.9
	192.7	123.7	259.2
	77.4–7507.2	2.6–319.9	38.0–1247.0

GCDCA (*μ*mol/L)	6209.0 ± 4347.8	3425.5 ± 3684.1	6739.6 ± 5380.0
	4021.1	1677.0	1616.3
	6132.0	2642.0	4995.0
	3027.3–10097.8	308.7–5746.8	2404.9–9474.5

GDCA (*μ*mol/L)	2476.4 ± 2803.8	1096.8 ± 1845.7	3244.3 ± 4605.7
	2593.1	840.1	1383.7
	1300.7	381.0	1110.5
	844.1–2995.0	8.9–1497.8	135.9–4421.3

GCA (*μ*mol/L)	8196.7 ± 6767.3	4639.1 ± 4891.7	5818.5 ± 4335.7
	6258.7	2226.7	1302.6
	6929.0	3335.0	5354.0
	2361.8–13126.0	709.1–6549.5	2794.8–7259.0

GHCA (*μ*mol/L)	22.8 ± 15.9	14.1 ± 17.6	43.8 ± 80.2
	14.7	8.2	24.1
	30.1	8.0	16.4
	7.7–36.3	2.7–14.6	10.3–33.8

TLCA (*μ*mol/L)	30.7 ± 35.2	19.4 ± 40.3	52.8 ± 75.0
	32.5	18.4	22.5
	22.3	5.2	19.3
	2.5–56.9	0–16.8	2.1–77.8

TUDCA (*μ*mol/L)	218.1 ± 454.6	72.9 ± 135.6	228.6 ± 474.5
	420.5	61.7	142.6
	46.8	19.2	58.9
	9.5–115.3	0–58.8	9.5–152.8

TCDCA (*μ*mol/L)	1837.6 ± 2285.3	1674.9 ± 1617.8	2324.9 ± 1758.2
	2113.5	736.4	528.2
	1118.5	1734.2	1689.3
	275.2–2301.4	282.3–2319.5	1113.4–3290.7

TDCA (*μ*mol/L)	603.8 ± 834.8	368.4 ± 655.1	786.4 ± 1136.1
	772.0	298.2	341.3
	278.8	118.3	377.5
	30.1–1100.8	7.1–321.6	79.8–930.7

TMCA (*μ*mol/L)	7.5 ± 9.5	7.2 ± 13.3	12.9 ± 22.5
	8.8	6.0	6.8
	4.5	3.4	5.9
	0–16.1	0–8.7	0–13.5

THCA (*μ*mol/L)	11.1 ± 16.3	28.3 ± 38.3	39.2 ± 71.5
	15.1	17.4	21.5
	3.8	11.1	12.1
	0.9–14.8	3.1–35.0	4.0–37.2

TCA (*μ*mol/L)	2183.6 ± 3050.8	2316.5 ± 2234.9	2443.0 ± 2141.9
	2821.6	1017.3	643.5
	1634.8	1658.5	1923.1
	366.1–1906.2	652.5–3343.5	1031.2–3048.0

CA (*μ*mol/L)	242.5 ± 280.7	16.6 ± 32.2	50.2 ± 145.5
	259.6	14.6	43.7
	97.8	0	2.6
	5.8–465.9	0–20.4	0–17.7

Total bile acids (mmol/L)	37.7 ± 23.6	68.4 ± 31.8	24.2 ± 15.9
	21.8	14.5	4.8
	26.7	65.8	21.9
	20.5–50.0	43.0–87.2	11.2–33.0

## Data Availability

The study data used to support the findings of this study have not been made available because of the General Data Protection Regulation (Regulation 2016/679 of the European Parliament and of the Council of 27 April 2016 on the protection of natural persons with regard to the processing of personal data and on the free movement of such data and repealing Directive 95/46/EC).

## Abbreviations

CA: Cholic acid
CDCA: Chenodeoxycholic acid
CE: Collision energy
C.I.: Confidence interval
CID: Collision-induced dissociation
DCA: Deoxycholic acid
ERCP: Endoscopic retrograde cholangiopancreatography
HCA: Hyocholic acid
HDCA: Hyodeoxycholic acid
LCA: Lithocholic acid
IQR: Interquartile range
IS-CAD_5_: Internal standard-D_5_-cholic acid
MCA: Muricholic acid
GCA: Glycocholic acid
GCDCA: Glycochenodeoxycholic acid
GDCA: Glycodeoxycholic acid
GHCA: Glycohyocholic acid
GHDCA: Glycohyodeoxycholic acid
GLCA: Glycolithocholic acid
GUDCA: Glycoursodeoxycholic acid
Std. Dev.: Standard deviation
TCA: Taurocholic acid
TCDCA: Taurochenodeoxycholic acid
TDCA: Taurodeoxycholic acid
THCA: Taurohyocholic acid
THDCA: Taurohyodeoxycholic acid
TLCA: Taurolithocholic acid
TMCA: Tauromuricholic acid
TUDCA: Tauroursodeoxycholic acid
UDCA: Ursodeoxycholic acid.
